# Stable Isotope and Signature Fatty Acid Analyses Suggest Reef Manta Rays Feed on Demersal Zooplankton

**DOI:** 10.1371/journal.pone.0077152

**Published:** 2013-10-22

**Authors:** Lydie I. E. Couturier, Christoph A. Rohner, Anthony J. Richardson, Andrea D. Marshall, Fabrice R. A. Jaine, Michael B. Bennett, Kathy A. Townsend, Scarla J. Weeks, Peter D. Nichols

**Affiliations:** 1 School of Biomedical Sciences, The University of Queensland, St Lucia, Queensland, Australia; 2 Climate Adaptation Flagship, CSIRO Marine and Atmospheric Research, Dutton Park, Queensland, Australia; 3 Manta Ray and Whale Shark Research Centre, Marine Megafauna Foundation, Praia do Tofo, Inhambane, Mozambique; 4 Biophysical Oceanography Group, School of Geography, Planning and Environmental Management, The University of Queensland, St Lucia, Queensland, Australia; 5 Centre for Applications in Natural Resource Mathematics, The University of Queensland, St Lucia, Queensland, Australia; 6 Wild Me, Praia do Tofo, Inhambane, Mozambique; 7 School of Biological Sciences, Moreton Bay Research Station, The University of Queensland, Dunwich, Queensland, Australia; 8 Wealth from Oceans Flagship, CSIRO Marine and Atmospheric Research, Hobart, Tasmania, Australia; Aristotle University of Thessaloniki, Greece

## Abstract

Assessing the trophic role and interaction of an animal is key to understanding its general ecology and dynamics. Conventional techniques used to elucidate diet, such as stomach content analysis, are not suitable for large threatened marine species. Non-lethal sampling combined with biochemical methods provides a practical alternative for investigating the feeding ecology of these species. Stable isotope and signature fatty acid analyses of muscle tissue were used for the first time to examine assimilated diet of the reef manta ray *Manta alfredi*, and were compared with different zooplankton functional groups (i.e. near-surface zooplankton collected during manta ray feeding events and non-feeding periods, epipelagic zooplankton, demersal zooplankton and several different zooplankton taxa). Stable isotope δ^15^N values confirmed that the reef manta ray is a secondary consumer. This species had relatively high levels of docosahexaenoic acid (DHA) indicating a flagellate-based food source in the diet, which likely reflects feeding on DHA-rich near-surface and epipelagic zooplankton. However, high levels of ω6 polyunsaturated fatty acids and slightly enriched δ^13^C values in reef manta ray tissue suggest that they do not feed solely on pelagic zooplankton, but rather obtain part of their diet from another origin. The closest match was with demersal zooplankton, suggesting it is an important component of the reef manta ray diet. The ability to feed on demersal zooplankton is likely linked to the horizontal and vertical movement patterns of this giant planktivore. These new insights into the habitat use and feeding ecology of the reef manta ray will assist in the effective evaluation of its conservation needs.

## Introduction

Information on the diet and trophic position of an animal can improve ecological understanding of the underlying drivers of its movements and its role within the ecosystem. Such knowledge can also support conservation plans for areas where the temporal and spatial abundance and distribution of prey are understood [1]–[3]. Stomach content analysis is the conventional approach used to assess a species’ diet [4] and has many advantages; however, it also has several shortcomings. First, this technique only provides a ‘snapshot’ of recent feeding and may not accurately reflect the composition of prey items that contribute most significantly to its general diet. This technique my also not necessarily account for ontogenetic or seasonal shifts in diet nor regional variability in the diet of a species. For a comprehensive understanding of a species’ diet, many specimens must be examined with samples from different seasons, locations, size classes and sexes. Sample collection therefore becomes challenging for widely distributed and wide-ranging species that may feed in numerous habitat types over large geographic areas. Second, stomach content analysis is heavily biased towards items resistant to digestion such as bones, exoskeletons, chelae and eyeballs [5]. Last, obtaining stomachs from large and threatened marine species is often difficult and killing animals for this purpose is ethically questionable.

The reef manta ray *Manta alfredi* (Krefft, 1868) is a large planktivorous elasmobranch with a circumglobal distribution in tropical and subtropical waters [6]. The species is listed as globally Vulnerable to extinction on the IUCN Red List of Threatened Species, mainly due to new or expanding targeted fisheries [7]. Many of these fisheries are considered unsustainable due to the relative small native population sizes, likely limited exchange between subpopulations and conservative life history of the species (i.e. slow growth rates, late age of sexual maturity, few offspring, and long life) [7], [8]. Although manta rays have gained considerable scientific attention over the past two decades and are heavily fished in several parts of the world [8], there is little information on their feeding ecology. The limited availability of stomach content samples for reef manta rays highlights the need for suitable alternative methods to study their diet. Biochemical approaches such as stable isotope (SI) and signature fatty acid(s) (FA) analyses can provide information on dietary preferences and trophodynamics in marine animals [9], [10]. Both techniques have the advantage of only requiring a small amount of tissue for analysis, which can be obtained as a biopsy from living animals with little impact on their welfare (e.g. [11], [12]).

Stable isotope analysis has been successfully used to examine aspects of the biology and ecology of several elasmobranch species [13]–[18]. Shifts in SI values of nitrogen (^15^N/^14^N or δ^15^N) and carbon (^13^C/^12^C or δ^13^C) in a consumer’s tissues are related to its assimilated food and provide an index of its relative trophic position in the ecosystem. δ^15^N and to a lesser extent δ^13^C show a predictable stepwise enrichment with each increasing trophic level [19]–[22]. The trophic position of a species can only be properly assessed with regional isotopic characterisation of the ecosystem as the baseline stable isotope data of the food chain may vary among regions [21]. The conservative fractionation of carbon between primary producers and consumers means that δ^13^C values provide information on the origin of the carbon entering the food web. δ^13^C values/signatures differ between benthic and pelagic habitats [23] and are influenced by marine, freshwater and terrestrial inputs [24]. However, there are caveats associated with the application of SI analysis in elasmobranchs. There is little information in the current literature on critical values such as diet-tissue discrimination factors and rate of isotopic incorporation rates, which are needed to interpret SI data correctly. Although a few studies on captive sharks have been undertaken [18], [25], [26], similar captive studies of many large elasmobranch, including manta rays, are impractical. Diet-discriminatory factors therefore need to be assumed from literature values of related species.

Signature FA analysis has been increasingly used to study the diet of a number of marine species including elasmobranchs [27]–[30]. In animals, FA are used as an energy supply, are stored in adipose tissue, and can play a structural role or be incorporated into specific metabolic pathways [9], [31], [32]. Selected FA are synthesised by higher consumers while others are generally assimilated intact, including the essential long-chain (≥C_20_) polyunsaturated fatty acids (LC-PUFA). Long-chain-PUFA in fish tissue are most likely to be derived directly from the diet as higher consumers generally lack the ability to biosynthesise these FA *de novo* [33], [34] and marine fishes are not likely to biosynthesise FA to a significant level due to their naturally PUFA-rich diet [34], [35]. Therefore, the FA signature profile of prey is likely to influence directly the FA profile of its consumer. Several LC-PUFA can also be used as biomarkers to trace the base of the marine food-web (e.g. diatoms and/or flagellate origin for marine phytoplankton) [33], [36].

Current knowledge of the feeding ecology of the reef manta ray is limited to one early and rudimentary description of one stomach content [37] and several field observations of foraging behaviour close to the surface at most known aggregation sites around the world (e.g. Australia [38]; Hawaii [39], Indonesia (*M. alfredi* but referred to as *M. birostris*) [40]; the Maldives [41], Mozambique (*M. alfredi* but referred to as *M. birostris*) [42] and the Central Pacific [43]). It has thus been presumed that manta rays feed predominantly on aggregations of near-surface zooplankton in productive coastal areas during the daytime. It is unknown, however, how important the observed surface feeding events are in terms of the total dietary intake of these large planktivores. In a pilot study, we reported the unusual FA profiles of the reef manta ray and the whale shark *Rhincodon typus* Smith 1828, both being dominated by omega-6 (ω6) PUFA [44]. These results were surprising as FA profiles of marine animals, and crustacean zooplankton in particular, are generally dominated by omega-3 (ω3) PUFA [33], [45]. Origins of such a distinctive profile remain ambiguous but it suggests that the feeding ecology of planktivorous elasmobranchs is more complex than previously thought. The purpose of this study was to couple SI and FA analyses of reef manta ray tissue, their known food, the near-surface zooplankton, and other potential prey items to provide a more comprehensive insight into their dietary ecology.

## Materials and Methods

### Ethics Statement

This study was conducted with permits from the GBR Marine Park Authority (G09/29853.1) and approval from the University of Queensland Animal Ethics Committee (SBMS/206/11/ARC).

### Samples Collection

#### Biopsy samples

Muscle and/or skin tissue samples were collected from the ventro-posterior area of the pectoral fins of free swimming reef manta rays, using a biopsy needle mounted on a modified Hawaiian hand-sling. Samples were collected from three aggregation sites for reef manta rays in waters off: Lady Elliot Island (24°06′S 152°32′E) and North Stradbroke Island (27°25′S 153°32′E) in Queensland, Australia and Praia do Tofo (23° 52′S 35° 33′E) in Mozambique.

Muscle tissue and skin tissue for biopsies obtained in Australia were prepared separately for SI and FA analyses. Biopsy samples collected in Mozambique were used for FA analysis only, and comprised muscle tissue, although small remnants of skin may have been present in some samples.

All samples were initially kept on ice and then stored at −20°C until required for analysis. Of the 22 reef manta ray biopsies collected in east Australia, 16 were from females and six from males. Of the 12 reef manta rays biopsies obtained from Mozambique, nine were from females and three from males.

#### Near-surface zooplankton

A total of 62 zooplankton samples were collected: 54 samples were from eastern Australia and 9 from southern Mozambique. In Australia, samples were collected from the upper 5 m of the water column by towing a 200 µm mesh size plankton net against the tidal current for 5 min at ∼2–4 knots. In Mozambique, a similar plankton tow was performed with a 200 µm mesh net or with a small 100 µm mesh hand-held net towed by a swimmer. Samples were kept on ice and processed (filtered and divided) on the same day. All samples were then frozen at −20°C. *Australia:* Two categories of zooplankton hauls were conducted to detect changes in the qualitative properties of the near-surface zooplankton that may influence the feeding activity of manta rays: 1) Feeding, when tows were collected within the feeding manta ray trail (n = 32); and 2) Not feeding, where tows were collected when reef manta rays were present but not feeding (n = 22). Samples were collected at Lady Elliot Island (n = 51) in June 2010, October 2010, February 2011, June 2011, August 2011, September 2011 and February 2012. For a greater diversity of samples, a few samples (‘not feeding’) were also included from North Stradbroke Island, 380 km further south and another reef manta ray aggregation site, from December 2011 (n = 2) and January 2012 (n = 1) when feeding can occur. Each sample was filtered and divided into four subsamples of which two were frozen at −20°C as soon as possible for subsequent FA and SI analyses. When large and obvious zooplankton species (e.g. gelatinous zooplankton, large copepods, chaetognaths, shrimp larvae) were abundant, several individuals of the same group were extracted from the fresh samples and frozen to be analysed separately for FA and SI. Representative specimens of each group were also fixed in formalin for subsequent taxonomic identification. Extracted taxa were classified into one of seven taxonomic groups: the calanoid copepod *Undinula vulgaris* (Dana 1849) (n = 11 samples), the calanoid copepod *Candacia ethiopica* (Dana 1849) (n = 3 samples), the calanoid copepod *Subeucanlanus* spp. (n = 1 sample), decapod crab larvae (n = 3 samples), shrimp-like larvae (n = 7 samples), fish larvae (n = 8 specimens) and eel larva (n = 1 specimen). Each sample comprised several specimens of the same category.

*Mozambique:* Near-surface zooplankton samples from Mozambique comprised three samples collected during reef manta ray feeding events and six samples collected when reef manta rays were not sighted in November and December 2011.

#### Epipelagic zooplankton

*Australia:* Six zooplankton samples were collected in waters 100 m deep off North Stradbroke Island. Vertically integrated hauls using a 200 µm mesh drop net were performed to collect zooplankton samples from between 75 and 82 m depth (n = 3). In addition, deep-horizontal plankton tows using a 200 µm mesh net were conducted at ∼20 m depth for 5 min within the same area (n = 3).

*Mozambique*: Three zooplankton samples were collected in ∼300 m deep water off the continental shelf ∼15 km east of Praia do Tofo. Vertically integrated hauls using a 200 µm mesh size net were performed at 50, 100 and 200 m depths.

#### Demersal zooplankton

Demersal (also known as emergent) zooplankton live within or close to the sea bottom and undergo daily vertical migration, emerging at night in high density [46], [47]. Although it is unknown whether reef manta rays were feeding at night and within the same area at Lady Elliot Island, it is plausible that these planktivores feed on demersal zooplankton, such as observed in Hawai’i [48]. An emergence trap using a 200 µm mesh net, based on the design of Alldredge & King [47] and Melo *et al.* [49], was secured to the sea-floor at a depth of 15 m and 8 m in waters adjacent to Lady Elliot Island prior to sunset and left in place overnight. Zooplankton that emerged from the substrate overnight were caught and retrieved the next morning. Five separate samples were collected and all were analysed for FA composition only due to limited amount available.

#### Regional isotopic characterisation for eastern australia

Muscle tissue was collected from the lateral fillet of two coastal teleost species: sea mullet *Mugil cephalus* Linnaeus, 1758 (n = 20) and stout whiting *Sillago robusta* Stead, 1908 (n = 20) caught by commercial vessels operating in southeast Queensland coastal waters. Isotopic values of muscle tissue from other pelagic fishes and elasmobranchs sampled in southeast Queensland were obtained from Revill *et al.* [50].

### Stable Isotope Analysis

Samples were soaked in distilled water for 15 min, rinsed and then oven-dried at 60°C for 24–48 h. Dried tissue (0.5–1.5 mg) was weighted into 8×5 mm tin capsules (SerCon p/n SC0009). All samples were analysed at the Stable Isotopes Analysis Lab, Australian Rivers Institute at Griffith University, Australia. Samples were combusted in a Sercon Europa EA-GSL elemental analyser (Sercon Ltd, UK). The resulting N_2_ and CO_2_ gases were chromatographically separated and analysed using Sercon Hydra 20–22 isotope ratio mass spectrometer (Sercon Ltd, UK).

Isotopic values are expressed using the standard δ notation, as part per thousand (‰) deviation from a standard. Stable isotope abundances were calculated using the equation:
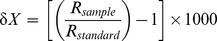
where *X* = ^15^N or ^13^C, *R* = the ratio ^13^C/^12^C or ^15^N/^14^N, with IAEA N1 and IAEA N2 as reference for nitrogen and IAEA-CH-6 for Carbon. All standards are traceable to atmospheric N_2_ and Vienna PeeDee Belemnite (VPDB) carbon respectively [51]–[52].

#### Lipid effect

Lipids are known to be depleted in ^13^C, resulting in lower δ^13^C values for tissue with greater lipid content. A high C: N ratio is indicative of high lipid content in aquatic organisms and lipid extraction or correction using a lipid normalisation model is recommended for tissue with C: N >3.5 [53]. The following lipid normalisation models were applied whenever appropriate to correct δ^13^C values for lipid effect:
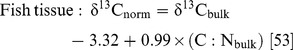


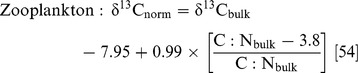
where δ^13^C_norm_ is the value of δ^13^C after lipid normalisation, δ^13^C_bulk_ is the direct measurement of δ^13^C of the target organism and the ratio C: N_bulk_ is calculated from direct measurement of C and N.

Enrichment values between reef manta rays and known prey were calculated using the equation:
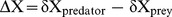
Where δX is δ^13^C or δ^15^N.

#### Trophic position

The trophic position (TP) of the reef manta ray was estimated using the following equation:
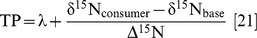
where λ is the trophic position of the selected consumer used to estimate δ^15^N_base_, δ^15^N_consumer_ is the direct δ^15^N value of the target species, δ^15^N_base_ is the δ^15^N value of a primary consumer for the local food web and Δδ^15^N is the trophic fractionation of δ^15^N per trophic level. The δ^15^N values of the known herbivorous calanoid copepod *Undinula vulgaris* (n = 6) collected at Lady Elliot Island were used as δ^15^N_base_. For accurate estimation of the trophic position of a species within the food web, species-specific knowledge on diet tissue discrimination factors (Δ^15^N = δ^15^N_consumer_ − δ^15^N_prey_) needs to be determined in a controlled environment. Since Δ^15^N has not been determined for any planktivorous elasmobranch, the discrimination factor from the leopard shark *Triakis semifasciata* Girard, 1855 of Δ^15^N = 3.7‰ for muscle tissue as determined by Kim *et al.* [18] was applied. This discrimination factor is the first value to be rigorously determined in laboratory controlled environment for elasmobranchs.

### Fatty Acid Analysis

#### Lipid extraction

Wet weight of each reef manta ray tissue and zooplankton sample was determined prior to analysis. Lipids were extracted overnight using the modified Bligh & Dyer [55] method with a one-phase methanol:chloroform:water (2∶1∶0.8 by volume) extraction. Phases were separated by adding water and chloroform leading to a final ratio of 1∶1∶0.9 methanol:chloroform:water and the lower chloroform phase containing the lipids was retained. Lipids were recovered by rotary evaporation of the chloroform *in vacuo* at ∼40°C. Total lipid extracts were concentrated in tared glass vials by application of a stream of inert nitrogen gas and weighed. Samples were then stored in chloroform at −20°C prior to further analysis.

#### Lipid classes

The total lipid extract from each sample was spotted on chromarods that were developed for 25 min in a polar solvent system (hexane:diethyl-ether:acetic acid, 60∶17∶0.1 by volume). Chromarods were then dried in an oven for 10 min at 100°C and analysed immediately. Lipid class composition was determined for each sample using an Iatroscan Mark V TH10 thin layer chromatograph combined with a flame ionisation detector. For comparison purposes, a standard solution containing known quantities of wax esters, triacylglycerols (TAG), free FA (FFA), sterols (ST) and phospholipids (PL) was run with the samples. Each peak was identified by comparison of Rf with the standard chromatogram. Peak areas were measured using SIC-480II Iatroscan™ Integrating Software v.7.0-E (System Instruments Co., Mitsubishi Chemical Medicine Corp., Japan) and quantified to mass per µl spotted using predetermined linear regressions.

#### Fatty acids

An aliquot of the total lipid extract was treated with 3 ml of a solution of methanol:hydrochloric acid:chloroform (10∶1∶1), heated at ∼80°C for 2 h. After cooling and addition of MilliQ water, the resulting FA methyl esters were extracted into hexane:chloroform (4∶1). Samples were dried under a stream of nitrogen gas before adding a C19 internal injection standard solution. Samples were then analysed using an Agilent Technologies 7890B gas chromatography (GC) (Palo Alto, California, USA) equipped with a non-polar Equity™-1 fused silica capillary column (15 m×0.1 mm i.d., 0.1 µm film thickness), a Flame Ionisation Detector, a split/splitless injector and an Agilent Technologies 7683 B Series auto sampler. Helium was the carrier gas. Samples were injected in split-less mode at an oven temperature of 120°C. After injection, oven temperature was raised to 270°C at 10°C.min^−1^ and finally to 300°C at 5°C.min^−1^. Peaks were quantified with Agilent Technologies ChemStation software (Palo Alto, California, USA). GC results are typically subject to an error of up to ±5% of individual component area. Peak identities were confirmed with a Finnigan ThermoQuest GCQ GC mass-spectrometer (GC-MS) system (Finnigan, San Jose,CA) [56]. The percentage for each FA was converted from the area of chromatogram peaks. All FA are expressed as percentage of total FA.

### Data Analyses

#### Stable isotopes

One-way ANOVA was used to test for statistical differences in stable isotopes values (δ^13^C and δ^15^N) between Lady Elliot Island and North Stradbroke Island using R v2.12.2 [57].

#### Fatty acids

Fatty acids were coded as A: B ωD, where A is the number of carbon atoms, B is the number of double bonds in the carbon chain and ωD is the position of the first double bond from the terminal methyl end of the molecule. Fatty acids were categorised as saturated (SFA), monounsaturated (MUFA) and polyunsaturated (PUFA), and each FA expressed as a percentage of the total FA (%TFA). Data are shown as mean ± standard error %TFA. ANOVA and ANOSIM were used to test for significant difference among samples. Pairwise ANOSIM was performed to identify the level of significant difference among the different groups in terms of FA composition. Due to the small sample size, interpretation of ANOSIM-*R* value was also used to evaluate the level to which groups differed, with *R* values >0.75 indicating clear separation among groups, *R* = 0.75–0.25 indicating separate groups with overlapping values and *R* <0.25 as barely separated groups [58]. All FA detected above trace levels (>0.2%) were used for within-group comparison, while all FA >1% were used for among-groups comparison [groups = reef manta rays (muscle and skin) and zooplankton: near-surface (feeding and non-feeding), epipelagic, demersal, zooplankton taxa]. Data were not transformed to avoid giving more weight to FA present in small quantities. SIMPER was used to identify the contribution of each FA to similarities within a group and to dissimilarities amongst different groups. Non-metric multi-dimensional scaling (MDS) plots were used to visualise groupings within and among reef manta rays, their known prey and other zooplankton collected. ANOSIM, SIMPER and MDS were generated using PRIMER v6 (Primer-E, UK) [58].

## Results

### Stable Isotopes

#### Prey and predators in east australia

There was no significant difference between reef manta ray muscle tissue from Lady Elliot Island and North Stradbroke Island for both δ^13^C and δ^15^N values (ANOVA, p>0.05). The mean δ^13^C and δ^15^N values for reef manta ray muscle tissue were −17.4±0.1‰ and 8.9±0.3‰ respectively (n = 12). Skin tissue samples (n = 6) were analysed separately and had mean δ^13^C value of −14.6±0.1‰ and δ^15^N value of 8.9±0.5‰ (Table 1). No lipid normalisation model was applied to δ^13^C values as the C: N ratio of all samples was <3.5. The estimated trophic position of reef manta rays was 3 (secondary consumer).

**Table 1:** Isotopic values (mean ± standard error) of main species analysed.

Species	Common name/notes	n	δ^13^C (‰)	δ^13^C (‰) normalised	δ^15^N (‰)
*Manta alfredi* (muscle)	Reef manta ray	13	−17.4±0.1	na	8.9±0.3
*Manta alfredi* (skin)	Reef manta ray	7	−14.6±0.1	na	8.9±0.5
*Undinula vulgaris*	Calanoid copepod (herbivore)	6	−20.3±0.1	−20.0±0.2	5.2±0.4
*Candacia ethiopica*	Calanoid copepod (carnivore)	2	−20.4±0.4	−20.4±0.4	7.4±0.1
Decapods	Crab larvae	3	−20.1±0.5	−18.4±0.5	4.6±0.6
Shrimp-like		5	−20.3±0.3	−19.6±0.4	5.8±0.5
Fish larvae	Fish larvae	5	−19.7±0.3	−19.7±0.2	5.7±0.2
Zooplankton tows	Feeding events only	32	−20.2±0.1	−18.7±0.1	6.5±0.2
*Mugil cephalus*	Sea mullet	20	−19.6±0.9	−19.8±0.9	10.2±0.7
*Sillago robustus*	Stout whiting	20	−17.4±0.1	na	11.7±0.1

All near-surface zooplankton samples collected were analysed for stable isotope composition, along with 21 samples of separate species/taxonomic groups (Table 1). The mean δ^13^C value for all zooplankton tows (n = 54) was −20.2±0.1‰ from direct measurements and −18.5±0.2‰ after applying the lipid normalisation model (C: N ratio for all samples ranged between 3.6–7.9). The mean δ^15^N value was 6.4±0.2‰. There was no significant difference between isotopic values of plankton collected during reef manta ray feeding events and non-feeding events (ANOVA p>0.05); however, only values of ‘feeding’ zooplankton samples were used for predator-prey comparison purposes. On average, reef manta ray muscle was enriched in ^13^C by 1.3‰ based on corrected δ^13^C values, and in ^15^N by 2.4‰ relative to the zooplankton sampled from feeding events.

#### Isotopic characterisation of eastern australia fishes

**Figure 1:**
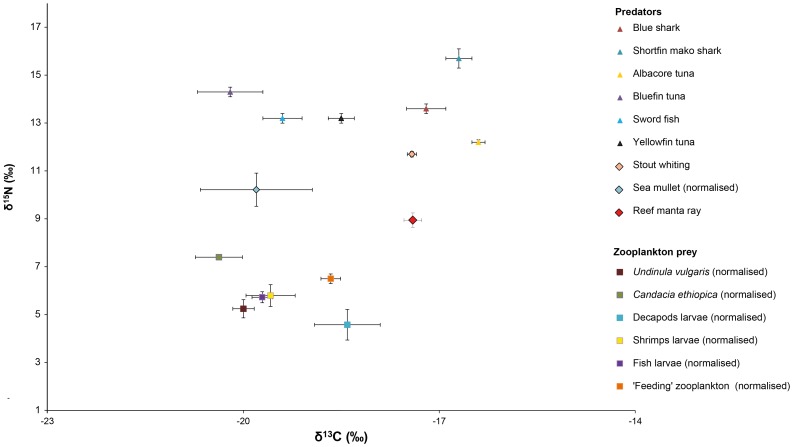
δ^15^N and δ^13^C values of zooplankton, pelagic predators and reef manta rays from southeast Queensland waters. Different symbols and colours indicate the mean isotopic values of different groups and species. All zooplankton values are adjusted to account for lipid normalisation based on Post [53]. Pelagic predator values (indicated by triangle icons) are based from Revill *et al.* [50]. Error bars represent standard error.

Mean δ^13^C and δ^15^N values were −17.4±0.1‰ and 11.7±0.1‰ respectively for stout whiting, and −19.6±0.9‰ and 10.2±0.7‰ respectively for sea mullet (Table 1, Figure 1). The lipid normalisation model for fish tissue was applied to δ^13^C values of sea mullets as the mean C: N ratio was >3.5 and the resulting mean δ^13^C normalised value was −19.8±0.9‰.

### Lipid and Fatty Acid Composition

Lipid class profiles of the reef manta ray were dominated by PL (80.8±1.7% of total lipids), followed by ST (10.3±0.9%). Lipids from all zooplankton samples collected in Australia were dominated by PL (ranging between 56–74%) and TAG (18–47%) (Table S1). Lipid classes in surface zooplankton collected in Mozambique were dominated by FFA (57.2%) followed by PL (30.2%), while epipelagic zooplankton collected within the same region showed a slightly higher contribution of PL (42.7%), followed by FFA (40.4%) (Table S1).

Of the 68 FA identified from all samples, 39 were above trace levels in reef manta rays (33 in both Australia and Mozambique) and 43 in zooplankton samples (Tables 2 & 3).

**Table 2:** Fatty acid composition (mean ± standard error %of total FA) for tissue biopsies of the reef manta ray *Manta alfredi* collected off eastern Australia and Mozambique.

Fatty acids	Australia (overall)	Australia (muscle)	Australia (skin)	Mozambique
	*n = 18*	*n = 15*	*n = 3*	*n = 12*
14∶0	0.3±0.1	0.3±0.1	0.1±0.1	0.4±0.1
16∶0	13.5±0.4	13.5±0.4	13.4±1.4	12.6±0.5
17∶0	0.6±0.1	0.5±0.0	1.1±0.1	0.6±0.1
18∶0	16.1±0.3	16.0±0.3	16.6±0.8	15.9±0.7
20∶0	0.4±0.0	0.3±0.0	0.7±0.1	0.4±0.1
22∶0	0.4±0.1	0.3±0.0	0.9±0.2	0.4±0.1
23∶0	0.1±0.0	0.1±0.0	0.3±0.1	0.2±0.1
24∶0	0.2±0.0	0.2±0.0	0.5±0.2	0.3±0.1
26∶0	0.3±0.1	0.3±0.1	0.1±0.0	0.0±0.0
**ΣSFA**	**32.0**±**0.5**	**31.6±0.6**	**33.9±0.7**	**31.0**±**0.9**
16∶1ω9c	0.1±0.0	0.1±0.0	0.5±0.2	0.3±0.1
16∶1ω7c	2.7±0.3	3.0±0.3	1.3±0.3	2.2±0.3
17∶1ω8c+a17∶0	0.5±0.0	0.4±0.0	0.8±0.1	0.7±0.1
18∶1ω9c	15.4±0.6	16.0±0.4	12.7±3.4	14.3±0.4
18∶1ω7c	6.3±0.3	6.8±0.3	3.9±0.4	5.7±0.4
19∶1	0.3±0.0	0.2±0.0	0.4±0.0	0.2±0.0
20∶1ω9c	1.2±0.1	1.2±0.1	1.4±0.2	1.1±0.0
20∶1ω7c	0.5±0.0	0.5±0.0	0.3±0.0	0.5±0.1
22∶1ω11c	0.2±0.1	0.2±0.1	0.1±0.1	0.2±0.1
22∶1ω9c	0.2±0.0	0.2±0.0	0.4±0.0	0.3±0.0
22∶1ω7c	0.6±0.0	0.6±0.1	0.6±0.1	0.7±0.1
24∶1ω9c	1.4±0.1	1.2±0.1	2.3±0.5	1.3±0.1
24∶1ω7c	0.2±0.0	0.2±0.0	0.3±0.1	0.2±0.0
26∶1ω7c	1.1±0.2	0.7±0.1	2.8±0.5	0.0±0.0
**ΣMUFA**	**31.0**±**0.8**	**31.4±0.7**	**28.8±3.6**	**28.1**±**0.8**
18∶2ω6	1.0±0.1	0.8±0.1	1.7±0.1	0.0±0.0
20∶4ω6	8.7±0.4	8.7±0.5	8.8±0.7	14.2±0.8
20∶5ω3	1.8±0.3	1.6±0.1	2.7±1.6	1.0±0.1
20∶3ω6	0.3±0.0	0.3±0.0	0.2±0.0	0.3±0.0
20∶2ω6	0.2±0.0	0.2±0.0	0.3±0.1	0.3±0.1
22∶5ω6	3.0±0.3	3.3±0.2	1.5±0.2	4.1±0.5
22∶6ω3	12.5±0.6	13.0±0.5	9.8±1.9	10.3±0.6
22∶4ω6	3.8±0.3	4.1±0.3	2.2±0.4	7.0±0.6
22∶5ω3	2.8±0.4	2.4±0.1	5.2±1.8	2.1±0.1
C22PUFA	0.4±0.1	0.4±0.1	0.5±0.2	0.0±0.0
26∶2	0.2±0.2	0.2±0.2	0.2±0.1	0.0±0.0
C28PUFA	0.3±0.1	0.3±0.1	0.8±0.1	0.0±0.0
**ΣPUFA**	**36.0**±**1.0**	**36.1±1.0**	**35.6±4.3**	**39.6**±**1.6**
ω3/ω6	1.0±0.07	1.1±0.1	0.9±0.3	0.55±0.05
Others	1.4±0.2	1.2±0.1	2.9±0.4	1.4±0.3
**Σ** *iso*-SAT+*anteiso*-SAT	1.1±0.1	0.9±0.1	1.7±0.2	1.1±0.3

**Σ**iso-SAT+anteiso-SAT = i15∶0, a15∶0, i16∶0, i17∶0, i18∶0.

others = 14∶1ω5c, 15∶0, C16PUFAs, 16∶1ω5c, 17∶1, C18PUFA, 18∶3ω6, 18∶4ω3, 18∶3ω3, 18∶1ω7t, 18∶1ω5c, 20∶4ω3, C20PUFA, 20∶1ω11c, 21∶5ω3, C22PUFA, 24∶5ω3, C24PUFA, 24∶1ω11c, C26PUFA, 26∶1ω9c, 26∶1ω7c.

Abbreviations: SFA- saturated fatty acids, MUFA- monounsaturated fatty acids, PUFA-polyunsaturated fatty acids.

**Table 3:** Fatty acid composition (% of total FA) of zooplankton samples collected off eastern Australia and Mozambique.

	Zooplankton mix (Australia)				Zooplankton mix (Mozambique)	
	Surface zooplankton (feeding)	Surface zooplankton (not feeding)	Epipelagic zooplankton	Emergent zooplankton	Surface zooplankton	Epipelagic zooplankton
*N*	*n = 29*	*n = 21*	*n = 6*	*n = 5*	*n = 9*	*n = 3*
14∶0	4.4±0.2	4.7±0.3	4.0±0.1	1.4±0.5	4.9±0.6	3.7±0.4
15∶0	0.7±0.0	0.8±0.0	1.1±0.1	0.6±0.1	0.6±0.0	1.1±0.1
16∶0	17.8±0.4	19.2±0.4	19.2±0.3	17.2±0.4	19.2±0.9	22.6±0.1
17∶0	1.2±0.1	1.3±0.1	1.7±0.1	2.0±0.3	1.4±0.1	2.0±0.0
18∶0	5.4±0.2	5.9±0.2	6.0±0.1	9.6±0.5	7.5±0.3	7.2±0.2
20∶0	0.5±0.0	0.4±0.0	0.4±0.0	0.7±0.1	0.5±0.1	0.8±0.1
22∶0	0.4±0.0	0.4±0.0	0.4±0.0	0.8±0.1	0.0±0.0	0.0±0.0
24∶0	0.4±0.0	0.4±0.0	0.4±0.0	0.4±0.1	0.7±0.2	0.5±0.0
26∶0	0.1±0.0	0.1±0.0	0.1±0.0	0.3±0.1	0.0±0.0	0.0±0.0
**ΣSFA**	**30.9±0.7**	**33.3±0.6**	**33.6±0.5**	**33.1±0.9**	**35.6±1.5**	**38.5±0.7**
16∶1ω7c	7.2±0.5	6.2±0.5	3.6±0.1	3.4±0.5	4.5±0.5	3.9±0.3
16∶1ω5c	0.3±0.0	0.2±0.0	0.1±0.0	0.1±0.0	0.2±0.0	0.1±0.0
17∶1ω8c+a17∶0	0.3±0.0	0.3±0.0	0.4±0.0	0.5±0.2	0.3±0.1	0.4±0.0
17∶1	0.0±0.0	0.1±0.0	0.2±0.0	0.5±0.2	0.1±0.0	0.3±0.1
18∶1ω9c	4.4±0.3	4.2±0.3	4.8±0.2	6.9±0.3	3.1±0.5	7.1±0.2
18∶1ω7c	2.8±0.2	2.4±0.1	2.2±0.1	4.6±0.5	1.9±0.2	2.7±0.0
19∶1	0.0±0.0	0.0±0.0	0.0±0.0	0.3±0.1	0.0±0.0	0.0±0.0
20∶1ω11c	0.2±0.0	0.2±0.0	0.2±0.1	1.5±0.7	0.2±0.1	0.4±0.2
20∶1ω9c	0.4±0.0	0.5±0.0	0.8±0.1	0.3±0.1	0.5±0.1	0.9±0.2
20∶1ω7c	0.3±0.0	0.2±0.0	0.2±0.0	0.3±0.1	0.2±0.0	0.2±0.0
22∶1ω11c	0.3±0.1	0.1±0.0	0.1±0.1	0.2±0.1	0.2±0.2	0.1±0.1
22∶1ω7c	0.3±0.0	0.2±0.0	0.1±0.0	0.1±0.0	0.3±0.1	0.0±0.0
24∶1ω11c	0.4±0.1	0.1±0.0	0.1±0.0	0.0±0.0	0.1±0.0	0.1±0.1
24∶1ω9c	1.4±0.1	1.2±0.1	0.9±0.0	0.2±0.1	1.7±0.1	1.3±0.1
24∶1ω7c	0.4±0.1	0.1±0.0	0.1±0.1	0.2±0.1	0.2±0.1	0.0±0.0
26∶1ω11c	0.2±0.1	0.2±0.0	0.4±0.1	0.8±0.1	0.0±0.0	0.0±0.0
26∶1ω7c	0.4±0.0	0.3±0.0	0.3±0.0	0.1±0.1	0.0±0.0	0.0±0.0
**ΣMUFA**	**20.0±0.9**	**17.1±0.7**	**14.9±0.6**	**20.7±1.7**	**14.1±1.8**	**17.7±**0.8
C16PUFAs	2.2±0.3	1.4±0.1	1.0±0.2	0.3±0.1	0.0±0.0	0.0±0.0
18∶3ω6	0.4±0.0	0.5±0.0	0.5±0.0	0.3±0.1	0.2±0.0	0.3±0.0
18∶4ω3	1.1±0.0	1.4±0.1	1.8±0.1	0.7±0.1	0.8±0.1	1.3±0.0
18∶2ω6	1.4±0.1	1.5±0.1	2.7±0.1	2.8±0.3	0.0±0.0	0.0±0.0
18∶3ω3	0.7±0.1	0.9±0.1	1.8±0.1	2.6±0.5	1.0±0.1	1.4±0.1
20∶4ω6	1.4±0.1	1.7±0.1	1.4±0.0	5.0±0.4	2.2±0.2	1.4±0.0
20∶5ω3	13.9±0.5	13.2±0.4	10.2±0.1	13.4±2.1	13.5±0.7	9.1±0.1
20∶3ω6	0.2±0.0	0.2±0.0	0.1±0.0	0.4±0.1	0.3±0.0	0.1±0.1
20∶4ω3	0.4±0.0	0.5±0.0	0.5±0.0	0.4±0.1	0.5±0.0	0.5±0.0
20∶2ω6	0.2±0.0	0.3±0.0	0.4±0.0	0.9±0.3	0.2±0.0	0.4±0.0
21∶5ω3	0.2±0.0	0.2±0.0	0.2±0.0	0.2±0.0	0.3±0.0	0.2±0.0
22∶5ω6	0.7±0.1	0.7±0.1	0.9±0.3	1.1±0.1	0.7±0.2	1.7±0.1
22∶6ω3	22.6±1.1	24.4±1.2	26.9±0.9	12.5±3.6	28.8±2.6	25.4±0.2
22∶4ω6	0.2±0.0	0.2±0.0	0.3±0.0	1.6±1.1	0.2±0.1	0.3±0.0
22∶5ω3	1.0±0.1	0.9±0.0	0.8±0.0	1.7±0.8	1.1±0.1	0.9±0.0
C22PUFAs	0.1±0.0	0.1±0.0	0.1±0.1	0.8±0.4	0.0±0.0	0.0±0.0
28∶5	0.9±0.1	0.6±0.0	0.8±0.1	0.3±0.1	0.0±0.0	0.0±0.0
**ΣPUFA**	**48.3±0.6**	**49.0±1.0**	**50.5±0.6**	**45.4±2.4**	**49.8±2.0**	**43.0±0.2**
ω3/ω6	9.5±0.5	8.7±0.6	6.9±0.5	2.9±0.6	12.8±2.0	9.3±0.1
Others	1.4±0.1	0.9±0.05	1.13±0.1	1.3±0.4	1.3±0.3	1.8±0.1
**Σ** *iso*-SAT+*anteiso*-SAT	0.8±0.0	0.7±0.0	0.8±0.1	0.8±0.2	0.3±0.1	0.3±0.1
EPA/DHA	0.6±0.04	0.5±0.04	0.4±0.02	1.1±0.2	0.5±0.1	0.4±0.0

**Σ**iso-SAT+anteiso-SAT = i15∶0, a15∶0, i16∶0, i17∶0, i18∶0.

others = 14∶1ω5c, 16∶1ω9c, C18PUFA, 18∶1ω7t, 18∶1ω5c, C20PUFA, C22PUFA, 22∶1ω9c, 23∶0, 24∶5ω3, C24PUFA, C26PUFA, 26∶2, 26∶1ω9c.

Abbreviations: SFA-saturated fatty acids, MUFA- monounsaturated fatty acids, PUFA-polyunsaturated fatty acids.

#### Reef manta ray tissue

Considering all FA above trace levels (n = 33), there was no significant difference among reef manta ray FA profiles from Lady Elliot Island and North Stradbroke Island (ANOSIM, *R* value = 0.056, p = 0.1). Thus, FA profiles from both locations were grouped as ‘Australia’ for further analysis. In addition, no significant difference was detected between sexes considering muscle tissue only (ANOSIM *R* value = −0.1, p = 0.7). There was a significant difference between muscle tissue (n = 15) and skin tissue (n = 3) FA profiles (ANOSIM *R* value = 0.9, p = 0.01). Average dissimilarity between the two tissues was 20% (SIMPER) and the three main contributors to differences were 18∶1ω9 (10.9%), docosahexaenoic acid (DHA, 22∶6ω3) (10.2%) and 22∶5ω3 (7.6%) (Table 2). In both tissue types, FA signatures were dominated by PUFA (36% TFA) and the main FA included 18∶0, 18∶1ω9, 16∶0, DHA and arachidonic acid (AA, 20∶4ω6) (Table 2), each contributing >8% to within-group similarity (Table S2). The PUFA profile of Australian reef manta rays showed similar levels of ω3 and ω6 PUFA, with a mean ω3/ω6 ratio of 1.1±0.1 for muscle tissue and 0.9±0.3 for skin tissue (Table 2). Docosahexaenoic acid was the main ω3 PUFA (13.0% in muscle tissue and 9.8% in skin tissue) while AA was the main ω6 PUFA (8.7% in muscle and 8.8% in skin tissue). Only low levels (<2%) of linoleic acid (LA, 18∶2ω6) were found in either tissue type (Table 2). All further comparisons were made using results from the muscle tissue of reef manta rays unless specified.

There was no significant difference in FA profile between male and female Mozambican reef manta ray tissues (ANOSIM *R* value = −0.1, p = 0.6). The FA profile of Mozambican reef manta rays was dominated by PUFA (38%TFA). The main FA included 18∶0, 18∶1ω9, AA, 16∶0 and DHA (Table 2), and each contributed at least 10% to within-group similarity (Table S2). Arachidonic acid was the main PUFA (14.2%) and DHA was the second highest PUFA (10.3%). Similar to Australian reef manta rays, only low levels (<1%) of LA were found.

**Figure 2:**
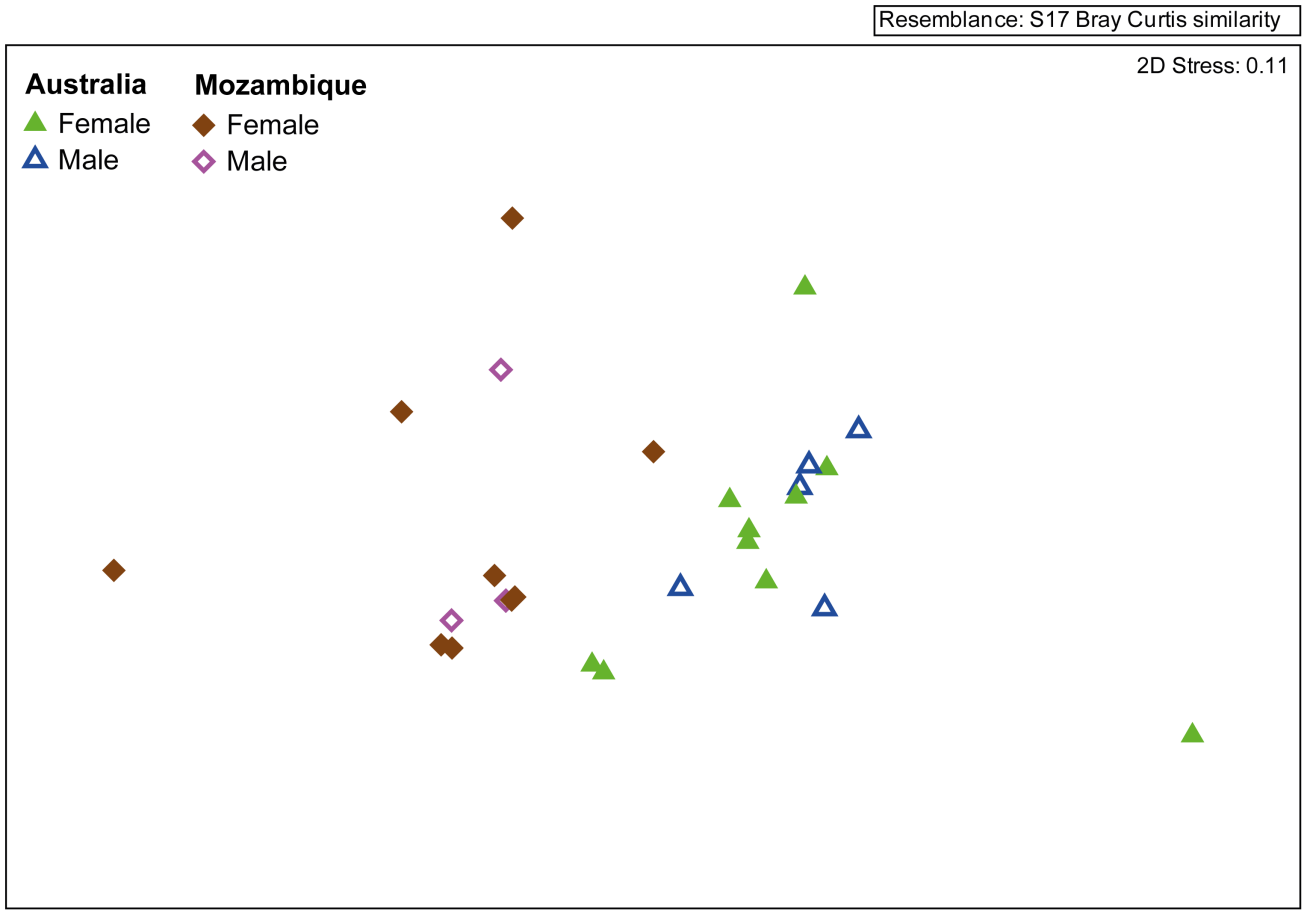
Regional comparison of reef manta ray muscle tissue fatty acid (FA) profiles. Multi-dimensional scaling ordinations of different sexes considering all FA >0.2% (n = 39).

Fatty acid profiles (considering all FA >0.2%, n = 39) of reef manta rays from Mozambique and Australia were significantly different from each other, but with a relatively high degree of overlap (ANOSIM, *R* value = 0.5, P = 0.001) (Figure 2). Average dissimilarity was 17% between the two regions (SIMPER). The PUFA profile of Mozambican reef manta rays was dominated by ω6 FA, with a mean ω3/ω6 ratio of 0.6 (Table 2), significantly lower than observed for the Australian reef manta rays (ANOVA, p<0.001). Mozambican reef manta rays had on average more ω6 than Australian reef manta rays. Arachidonic acid, DHA and 22∶4ω6 were the main contributors to dissimilarity between the two regions (SIMPER, 17.4%, 10.1% and 10.0% respectively), with higher levels of AA and 22∶4ω6 in Mozambican reef manta rays, and higher DHA levels in Australian reef manta rays (Table 2).

#### Near-surface zooplankton

There was no significant difference between near-surface zooplankton FA profiles (considering the main 41 FA >0.2%) collected during reef manta ray ‘feeding’ and ‘non-feeding’ events at Australian sites (ANOSIM, *R* value = −0.026, p = 0.7). All near-surface samples from Australia were PUFA-dominated with a mean of 48.6±0.5%, and DHA, 16∶0 and eicosapentaenoic acid (EPA, 20∶5 ω3) as major FA (Tables 3 & S2). Only low levels of the ω6 essential PUFA AA (1.5±0.09%) and LA (1.4±0.04%) were detected. The FA profile of near-surface zooplankton was largely dominated by ω3 PUFA with an overall mean ω3/ω6 ratio of 9.1±0.4 (Table 3). Docosahexaenoic acid was the dominant FA for all samples with a mean EPA/DHA ratio of 0.6±0.03 and a mean 16∶1/16∶0 ratio of 0.4±0.02. Significant differences were found among the sampled months, with DHA being the main contributor to dissimilarities between most months (Figure S1).

There was no significant differences between zooplankton collected during reef manta ray feeding events and those collected when no manta ray were sighted in Mozambican waters (ANOSIM *R* value = 0.1, p = 0.3). All near-surface zooplankton from Mozambique was PUFA dominated (49.8±2.0%) with DHA, 16∶0 and EPA as the three main FA. They also had low levels of AA (2.2±0.2%) similar to the FA profile of samples from Australian waters (Tables 3). The FA profile was also ω3 PUFA-dominated, with a mean ω3/ω6 ratio of 12.8±2.0 and DHA was also the most abundant FA for all samples (Table 3).

The seven zooplankton groups extracted from feeding event samples collected in Australia were analysed separately for FA composition (Table S3). All groups were dominated by PUFA (42.1–60.9% TFA), DHA was the main FA in all samples and the ω3/ω6 ratio varied from 4–10.4 (Table S3). Arachidonic acid and LA were present at low relative levels, ranging between 1.4–3.0%.

#### Epipelagic zooplankton

Due to the low sample size, data from both vertical haul (n = 3) and deep tows (n = 3) conducted off North Stradbroke Island were grouped as epipelagic zooplankton for further analysis. No significant difference was detected between epipelagic zooplankton and near-surface zooplankton in Australia (ANOSIM *R* value = 0.005, p = 0.4). The FA profile of epipelagic zooplankton was very similar to that of near-surface zooplankton, being dominated by PUFA (50.5±0.6%TFA) with DHA as the main FA and the ω3/ω6 ratio was high with a mean of 6.9±0.5 (Table 3).

Similar to the Australian samples, there was no significant difference between near-surface and epipelagic zooplankton in Mozambican waters (ANOSIM *R* value = 0.27, p = 0.1). The FA profile of epipelagic zooplankton was comparable to that of near-surface zooplankton in being dominated by PUFA with DHA as the main FA and a high ω3/ω6 ratio of 9.3±0.1 (Table 3).

#### Demersal zooplankton

**Figure 3:**
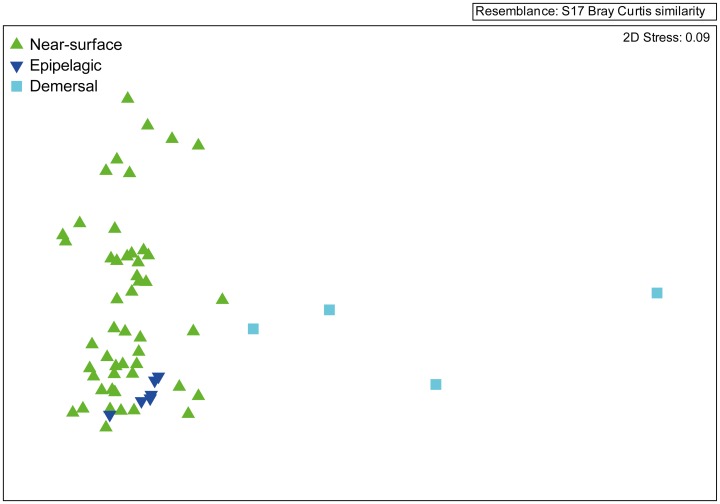
Comparison of zooplankton fatty acid (FA) profiles. Multi-dimensional scaling ordinations of zooplankton groups from different sampling areas collected in eastern Australia, considering all FA >0.2% (n = 50).

The FA profile of the five demersal zooplankton samples collected were significantly different and well separated from epipelagic zooplankton (ANOSIM, *R* value = 0.79, p>0.05) and near-surface zooplankton (ANOSIM, *R* value = 0.79, p = 0.01) of Australian waters (Figure 3). The major contributor to dissimilarities between groups was DHA in both comparisons (SIMPER, 22–30%), the second main contributors were EPA between demersal and epipelagic zooplankton (SIMPER, 7.8%) and 18∶0 between demersal and near-surface zooplankton (SIMPER, 6.9%). Arachidonic acid was the third contributor for both comparisons (SIMPER, 6.4–6.9%). Overall samples were dominated by PUFA (45.4%TFA) with EPA and DHA as the two main PUFA (Table 3). The FA 16∶0, EPA and 18∶0 contributed at least 10% to the within-demersal group similarities (Table S2). The ω3/ω6 ratio was lower than for near-surface zooplankton with a mean of 2.9±0.6. Arachidonic acid levels were also higher with a mean of 5.0±0.4% (Table 3).

#### Reef manta ray FA profiles in relation to zooplankton

Fatty acid profiles of Australian reef manta ray muscle tissue and zooplankton collected during feeding events at Lady Elliot Island were significantly different with no overlap between the two groups (considering 20 FA, ANOSIM, *R* value = 1, p = 0.01). Average percentage of dissimilarities between the two groups was 44.9% (SIMPER). Of the 20 FA compared, five contributed to >60% of the dissimilarities between the two groups: EPA (15.0%), 18∶1ω9c (13.6%), 18∶0 (13.2%), DHA (12.5%) and AA (9.1%). Reef manta rays had higher levels of 18∶1ω9, 18∶0 and AA while ‘feeding’ zooplankton had higher levels of EPA and DHA (Tables 2 & 3). Similar results were observed in Mozambique, where reef manta ray tissue was significantly different to near-surface zooplankton. The main FA contributing to >60% of dissimilarities were DHA (19.4%), EPA (13.2%), AA (12.6%), 18∶1ω9 (11.8%) and 18∶0 (8.9%), with an overall average dissimilarity of 51% (SIMPER). As for the Australian samples, 18∶1ω9, 18∶0 and AA were in higher relative proportion in reef manta rays and EPA and DHA were higher in near-surface zooplankton (Tables 2 & 3).

**Figure 4:**
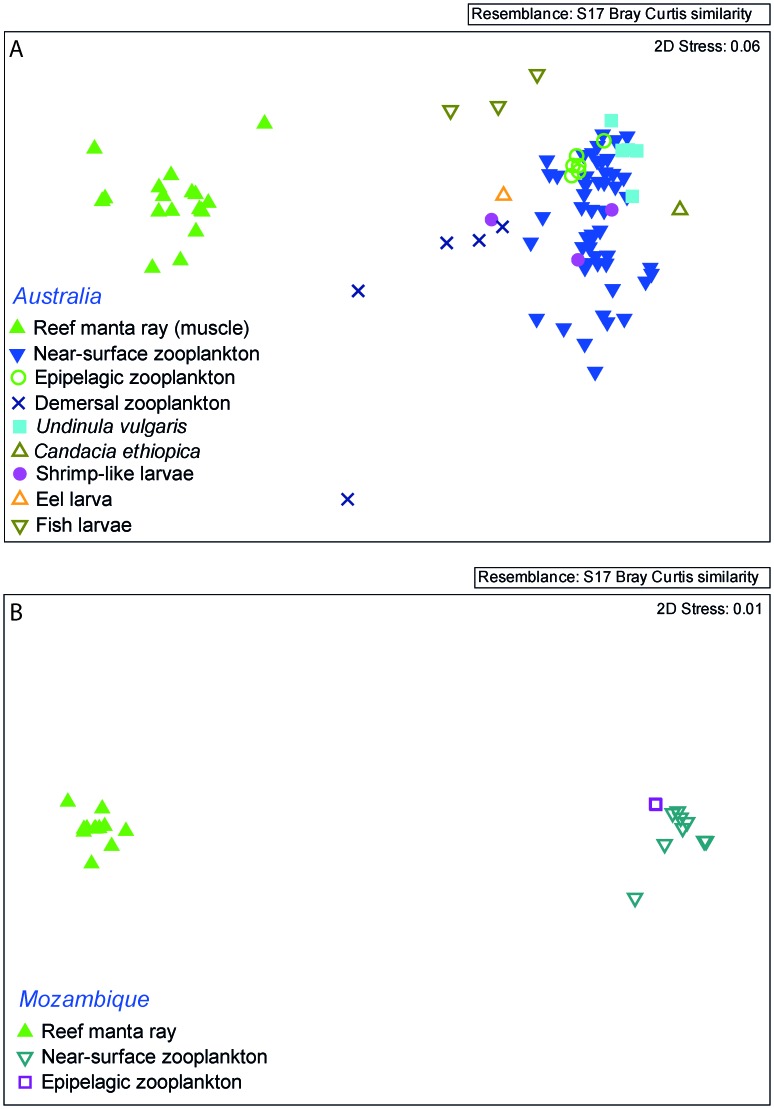
Comparison of reef manta ray tissue and zooplankton fatty acid (FA) profiles. Multi-dimensional scaling ordinations of (A) Australian reef manta ray muscle tissue and different zooplankton groups FA profiles collected off east Australia considering all FA >1% (n = 20), (B) Mozambican reef manta ray muscle tissue and different zooplankton groups collected off Mozambique considering all FA >1% (n = 20).

Reef manta ray FA composition was significantly different to all zooplankton types collected in this study for both Australia and Mozambique (Figure 4A–B), and all groups were well separated (ANOSIM *R* values≈1) for both the Australian and Mozambican samples. Average dissimilarities among reef manta rays and near-surface zooplankton, epipelagic zooplankton and all separated zooplankton taxa were between 42.2% and 51.0% (SIMPER). Average dissimilarity between Australian reef manta rays and demersal zooplankton was slightly lower with a value of 34.5%. The main contributors to dissimilarities between groups were EPA, DHA (both lower in reef manta rays), 18∶1ω9c, 18∶0 and AA (all higher in reef manta rays) (Tables 2, 3 & S3).

## Discussion

Reef manta rays have been presumed to feed predominantly on near-surface zooplankton, an assumption based primarily on field observations that are temporally and spatially limited to daytime and coastal areas [38], [40]. To our knowledge, no assessment of the food source of the reef manta ray is available in the scientific literature apart from the single stomach content rudimentarily described for *M. alfredi* (referred to as *Daemomanta alfredi*) by Whitley [37]. Here we present the first validation that reef manta rays feed on zooplankton with a new insight on the origin of their prey. The high site affinity individuals display to their aggregation sites over extended periods of time (>4 years [38], [59]) suggest that reef manta rays exploit food sources found within a same region (e.g. mid-eastern Australia). Reef manta rays are large vertebrates that most likely require a large amount of food to sustain their activities. Although manta rays are regularly observed feeding near the surface at aggregation sites, the food obtained from these temporary productivity blooms might not be enough to sustain these animals considering the large distances they seasonally travel (up to 500 km [38]). The results in this study suggest that reef manta rays do not feed predominantly on near surface zooplankton and that a major part of their diet may come from demersal sources.

Our findings demonstrate that the reef manta ray has a trophic level value of ∼3 indicative of a secondary consumer trophic position. The mean nitrogen enrichment value (Δ^15^N) between reef manta ray tissue and ‘feeding’ zooplankton of 2.4‰ falls within the range estimated of diet-tissue trophic fractionation in elasmobranchs (2.29–3.7‰) [18], [25]. This finding suggests that the nitrogen assimilated by reef manta rays originated from primary consumers, which is consistent with previous assumptions that the species feeds on zooplankton.

Highly depleted δ^13^C values are indicative of pelagic feeding (δ^13^C = −22‰ to ∼ −17‰ for pelagic phytoplanktonic origin [23]) while enriched values reflect more inshore and/or benthic foraging (δ^13^C >−17‰, for marine benthic algae [23]). The δ^13^C value of reef manta ray muscle tissue (−17.4‰) falls within the transition range between pelagic and inshore-benthic values, suggesting that their diet is not exclusively based on pelagic zooplankton. The δ^13^C value of reef manta ray skin tissue was more enriched compared to muscle tissue. Although differences in isotopic values between these two tissue types were found in other studies (e.g. [11]), there has been no experimental investigation on the origin of these variations. Yet, Pinnegar and Polunin [60] showed that teleost white muscle provided the best average of assimilated diet when compared to other tissue types. Interestingly, the stout whiting, that mostly feeds on benthic animals [61], [62], had a similar δ^13^C signature to the reef manta ray muscle tissue, which suggests that benthic organisms may form a part of the diet of reef manta rays.

The enrichment value of δ^13^C between reef manta ray muscle and zooplankton collected from feeding events appeared relatively high (1.3‰) when compared to other studies. Post [21] estimated that the carbon discriminator factor for teleost fishes was 0.4±1.3‰, while Hussey *et al.* [25] found a mean of 0.9±0.33‰ based on results for several elasmobranch species. Kim *et al.* [18] conducted the first study of elasmobranch stable isotopes under a fully controlled environment and found a discriminating factor of 1.7‰ for the leopard shark. However, this relatively high value is likely to be biased by the protein-rich diet the sharks were fed on, which led to enriched δ^13^C. Considering these previous studies, δ^13^C enrichment value for reef manta rays relative to their known prey is within the diet-tissue discrimination factor range for elasmobranchs, but is higher than average. This suggests that an important part of the diet of reef manta rays may be more carbon-enriched than the collected pelagic zooplankton. Pitt *et al.* [63] showed that emergent zooplankton is more enriched in carbon than pelagic zooplankton. Although these latter findings are limited to a coastal lagoon system, they provide some support for the suggestion that reef manta rays could feed on demersal zooplankton.

The difference in lipid class proportion between zooplankton from Australia and Mozambique was consistent with lower storage lipids in the Mozambican samples, and also showed degradation of the Mozambican samples, causing higher FFA levels. All zooplankton samples had relatively high levels of TAG that are usually associated with energy storage [64], [65]. Previous studies on chondrichthyan species showed that TAG tend to be stored in the liver [27], which may explain the low level of TAG found in reef manta ray muscle. As with other elasmobranch species [27], FA were mostly integrated as PL in reef manta ray muscle tissues which were high in PUFA. Dietary FA are selectively incorporated into different tissues and little is known about which tissue FA profile would best mirror the diet FA profile of elasmobranchs. Beckmann *et al.* [66] found that the liver of captive Port Jackson sharks *Heterodontus portusjacksoni* (Meyer, 1793) can reflect dietary FA within a short timescale (10 weeks) under controlled feeding experiments. McMeans *et al.* [35] showed that muscle FA profile in the Greenland shark *Somniosus microcephalus* (Bloch & Schneider, 1801) is highly representative of its prey FA profiles, and indicated that most FA undergo direct assimilation into this particular tissue. Although PL are less influenced by changes in diet than TAG [67], PL-rich muscle tissue can still provide an integrated diet signal over a longer period of time and it may be more representative for PUFA-rich prey items [27]–[29].

Near surface and epipelagic zooplankton in both Australia and Mozambique were largely dominated by ω3 PUFA, which is typical for most pelagic marine animals [33], [68]. All samples were DHA-dominated and low values of EPA/DHA and 16∶1/16∶0 (both <1) indicate a dominant flagellate-based diet in Australian and Mozambican pelagic zooplankton [33]{Dalsgaard, 2003 #24;Dalsgaard, 2003 #24}. Seasonal variation in the relative levels of DHA at Lady Elliot Island likely indicates temporal changes in available phytoplankton. Docosahexaenoic acid was the dominant PUFA in reef manta rays in east Australian waters and the second major PUFA in those from Mozambique, which likely reflect the intake of regional pelagic zooplankton in their diet. However, the high levels of ω6 PUFA for reef manta rays from both regions indicate that their diet is not restricted to ω6 PUFA-poor near-surface and epipelagic zooplankton. Although variations in FA compositions between muscle and skin tissues were detected in Australian reef manta rays, both tissue types provided similar relative proportions of assimilated FA and, had similar ω3/ω6 ratios. Results obtained from Mozambican reef manta rays (muscle and residual skin) are thus considered comparable to muscle tissue obtained in east Australia. The difference in FA profiles of reef manta rays from Australia and Mozambique could be due to more prominent foraging activity on ω6 PUFA-rich zooplankton by reef manta rays in Mozambique. The trophic pathway of high levels of ω6 PUFA in animals is still ambiguous. Whale sharks, which share many common life history traits with reef manta rays, also had unusually high levels of ω6 FA in their tissue, although whale sharks had much lower values of DHA than reef manta rays [30], [44]. Based on a range of comparative analyses of available FA profiles, together with modelled FA profiles and stomach content analysis, whale sharks were suggested to feed on demersal and deep-sea macrozooplankton and small fishes in addition to epipelagic zooplankton [30]. Demersal zooplankton from our study had relatively higher levels of ω6 PUFA compared to near-surface and epipelagic zooplankton. This corroborates previous studies that showed that benthic animals tend to have higher relative levels of ω6 PUFA and especially AA (e.g. [68]–[71]). Transfer of ω6 PUFA to demersal and benthic zooplankton could be through direct intake of ω6 PUFA-rich macroalgae [65], but also through the consumption of micro-heterotrophs that are present in the sediment and potentially feed on ω6 PUFA-rich phytodetritus [72]–[75].

**Figure 5:**
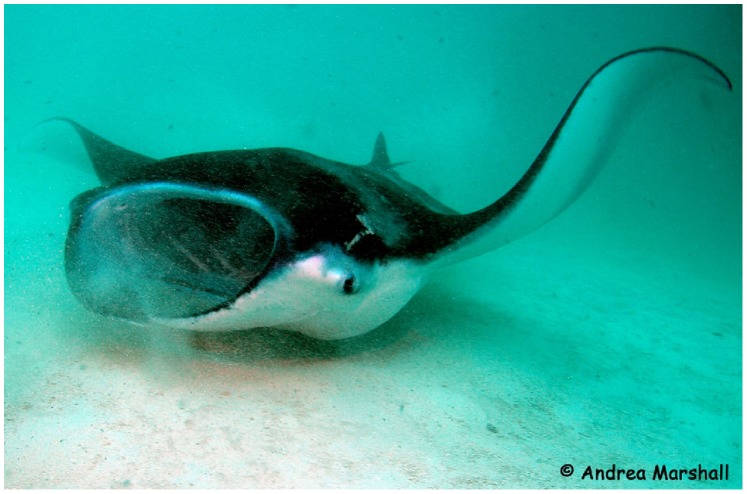
Reef manta ray feeding close to the sea bottom. This is occasionally observed during the day and proposed to be more common at night.

The zooplankton density threshold that may trigger foraging activity in reef manta rays is not known, but it is likely that these large planktivores target patches of high zooplankton density and biomass as has been shown for whale sharks and basking sharks *Cetorhinus maximus* (Gunnerus, 1765) [76]–[79]. Demersal zooplankton is highly abundant in shallow coastal areas and usually has larger individuals than pelagic zooplankton, leading to greater biomass [46], [47]. It is thus highly plausible that reef manta rays target demersal zooplankton when emerging from the sediment, especially at night. Consumption of this food source could explain the origin of the ω6 PUFA-rich profile of reef manta rays and their enriched δ^13^C values relative to values for pelagic zooplankton species. To date, studies that have focused on the FA or SI composition of demersal zooplankton in tropical and subtropical systems are scarce [63]. Behavioural observations at foraging sites revealed that reef manta rays can adapt their feeding strategy according to zooplankton distribution and individuals have been photographed feeding near the sea floor during the day (as illustrated in Figure 5). In addition, recent investigations of vertical movements of satellite-tracked reef manta rays in eastern Australia revealed that individuals commonly spend long periods of time at depth in the epipelagic zone, which could be associated with feeding activity in specific layers of the water column (FR Jaine, unpublished data).

Species-specific studies on a wide range of elasmobranch fishes are required to examine differences in biochemical composition between tissue types and determine how accurately the information provided by FA and SI analyses reflects a species’ diet. Muscle and skin tissues should be a major focus of study, since they can easily be collected as live-animal biopsies from large and mobile marine species, such as manta rays. Data on the vertical habitat use of reef manta rays from different regions, along with information on the local FA composition of different types of zooplankton (i.e. epipelagic, deep-sea, and in particular demersal/emergent) will help further resolve the role demersal zooplankton plays in the feeding ecology of reef manta rays. Our results suggest that future work should investigate potential food sources present in deeper waters, particularly in terms of their FA profiles and potential origins (e.g. benthic, upwelling origin, deep scattering layers).

Our findings challenge the prevailing paradigm on the feeding ecology of reef manta rays, and suggest that these large planktivores also feed on demersal and deeper-water zooplankton, and supplement their diet with opportunistic feeding in near-surface waters. A comprehensive knowledge of the foraging habits of the reef manta ray is necessary to identify the trophic and ecological role of the species and provide a broader understanding of its community dynamics. Identifying critical foraging habitats should help inform conservation management in areas where the species is most vulnerable.

## Supporting Information

Figure S1 **Comparison of zooplankton fatty acid (FA) profiles.** Multi-dimensional scaling ordinations of near- surface zooplankton FA profiles sampled at Lady Elliot Island from June 2010 to February 2012, considering all FA >0.2% (n = 41). There was a significant difference among samples (ANOSIM, *R* value = 0.74, p = 0.001) and pairwise comparison revealed that all sampled months were significantly different from each other (pairwise ANOSIM, p<0.05). Most groups were well separated with an *R* value >0.75. Some degree of overlap (ANOSIM, *R* value ranged between 0.50 and 0.75) was detected between June 2010 and June 2011, September 2011 and February 2011, and June 2010 and August 2011. A relatively high degree of overlap was found between June 2011 and August 2011 (*R* value = 0.4) and February 2011 and February 2012 (*R* value = 0.3). The three main FA contributing to discrimination of particular months were DHA, EPA and 16∶0 (SIMPER). The major contributor to dissimilarities between most months was DHA and it was the second main contributor in three cases, where either EPA (between June 2010 and August 2011) or 18∶1ω9 (between November 2010 and February 2012, October 2010 and February 2012) was the major contributor. All samples were dominated by DHA and 16∶0.
(TIF)

Table S1 **Lipid class composition (% of total lipids) and total lipid content (mg.g^−1^ of wet weight, ww) of (a) reef manta rays muscle tissue and (b) zooplankton samples.**
(DOCX)

Table S2 **Results of similarity percentage analysis (SIMPER) of fatty acid data for reef manta rays and zooplankton.** Fatty acids with an average contribution >8% are included. Data were not transformed prior to analysis
(DOCX)

Table S3 **Fatty acid composition (% of total FA) of zooplankton taxa collected off eastern Australia.**
(DOCX)
