# Supplementary figures and images for: Stable Isotope and Signature Fatty Acid Analyses Suggest Reef Manta Rays Feed on Demersal Zooplankton
Source: PLoS One. 2013 Oct 22;8(10):e77152. doi: 10.1371/journal.pone.0077152 (PMC3805558; doi:10.1371/journal.pone.0077152)

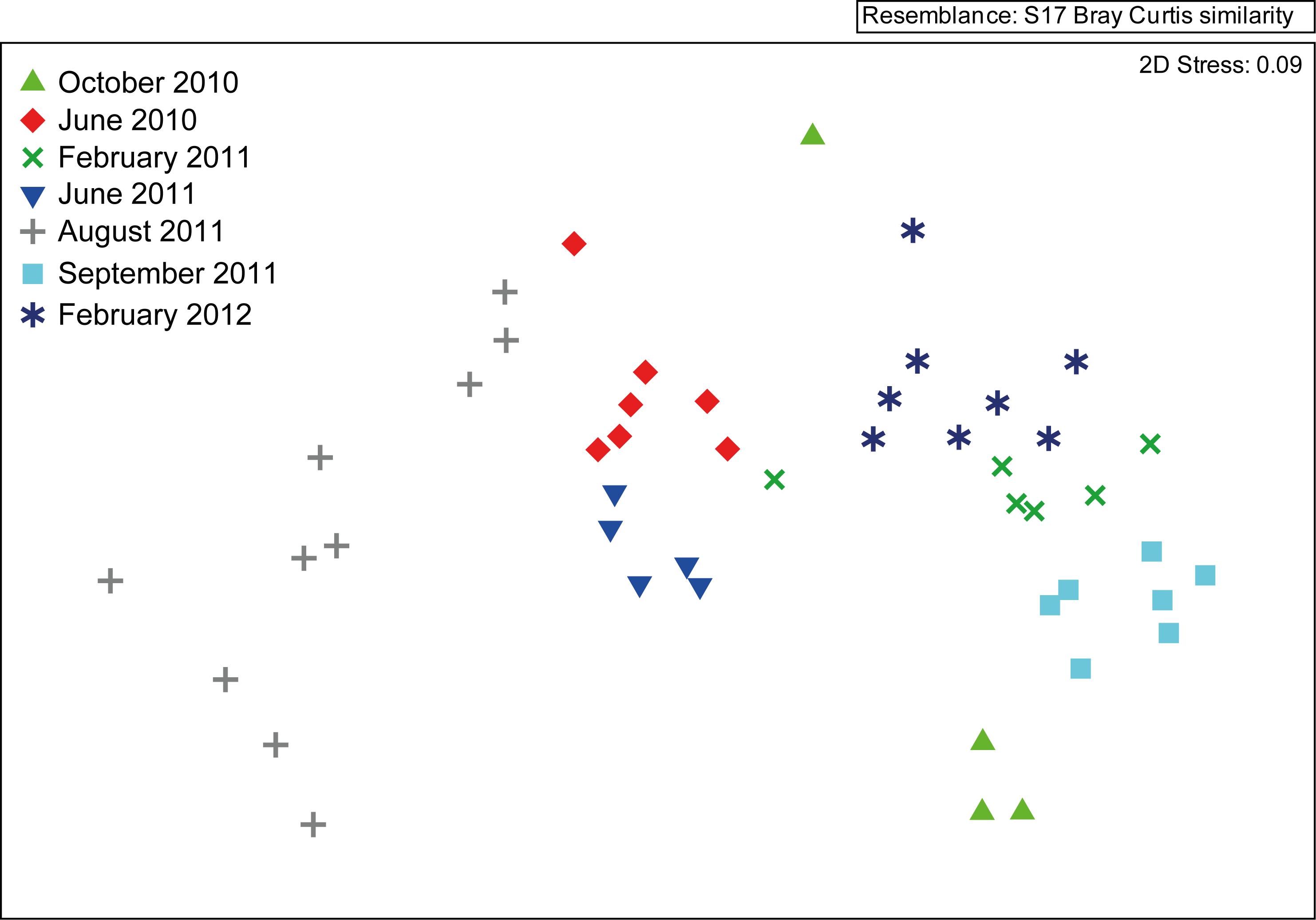

Supplement: Figure S1 — Comparison of zooplankton fatty acid (FA) profiles. Multi-dimensional scaling ordinations of near- surface zooplankton FA profiles sampled at Lady Elliot Island from June 2010 to February 2012, considering all FA >0.2% (n = 41). There was a significant difference among samples (ANOSIM, R value = 0.74, p = 0.001) and pairwise comparison revealed that all sampled months were significantly different from each other (pairwise ANOSIM, p<0.05). Most groups were well separated with an R value >0.75. Some degree of overlap (ANOSIM, R value ranged between 0.50 and 0.75) was detected between June 2010 and June 2011, September 2011 and February 2011, and June 2010 and August 2011. A relatively high degree of overlap was found between June 2011 and August 2011 (R value = 0.4) and February 2011 and February 2012 (R value = 0.3). The three main FA contributing to discrimination of particular months were DHA, EPA and 16∶0 (SIMPER). The major contributor to dissimilarities between most months was DHA and it was the second main contributor in three cases, where either EPA (between June 2010 and August 2011) or 18∶1ω9 (between November 2010 and February 2012, October 2010 and February 2012) was the major contributor. All samples were dominated by DHA and 16∶0. (TIF) [file pone.0077152.s001.tif]
