# Supplementary material for: Stable Isotope and Signature Fatty Acid Analyses Suggest Reef Manta Rays Feed on Demersal Zooplankton
Source: PLoS One. 2013 Oct 22;8(10):e77152. doi: 10.1371/journal.pone.0077152 (PMC3805558; doi:10.1371/journal.pone.0077152)
Supplement: Table S1 — Lipid class composition (% of total lipids) and total lipid content (mg.g−1 of wet weight, ww) of (a) reef manta rays muscle tissue and (b) zooplankton samples. (DOCX) [file pone.0077152.s002.docx]

Table S1. Lipid class composition (% of total lipids) and total lipid content (mg.g^-1^ of wet weight, ww) of (a) reef manta rays muscle tissue and (b) zooplankton samples.

| (a) |  | Reef manta ray | | | | | | | | | | | | | | | | | | | | | | | | | | |
| --- | --- | --- | --- | --- | --- | --- | --- | --- | --- | --- | --- | --- | --- | --- | --- | --- | --- | --- | --- | --- | --- | --- | --- | --- | --- | --- | --- | --- |
|  |  | overall (n=23) | | | | | |  | Australia muscle (n=14) | | | | | |  | Australia skin (n=3) | | | | | |  | Mozambique (n=6) | | | | | |
| Lipid class |  | %Total lipid | | | Content (mg/g) | | |  | %Total lipid | | | Content (mg/g) | | |  | %Total lipid | | | Content (mg/g) | | |  | %Total lipid | | | Content (mg/g) | | |
|  |  |  |  |  |  |  |  |  |  |  |  |  |  |  |  |  |  |  |  |  |  |  |  |  |  |  |  |  |
| WE |  | 2.1 | ± | 0.6 | 0.04 | ± | 0.02 |  | 1.3 | ± | 0.5 | 0.0 | ± | 0.0 |  | 8.1 | ± | 1.7 | 0.2 | ± | 0.1 |  | 1.0 | ± | 0.5 | 0.0 | ± | 0.0 |
| TAG |  | 3.9 | ± | 0.5 | 0.2 | ± | 0.03 |  | 3.2 | ± | 0.3 | 0.1 | ± | 0.0 |  | 6.5 | ± | 0.7 | 0.3 | ± | 0.0 |  | 4.1 | ± | 1.9 | 0.2 | ± | 0.1 |
| FFA |  | 2.9 | ± | 0.4 | 0.1 | ± | 0.01 |  | 2.4 | ± | 0.5 | 0.1 | ± | 0.0 |  | 5.4 | ± | 0.4 | 0.2 | ± | 0.0 |  | 3.0 | ± | 0.4 | 0.1 | ± | 0.0 |
| ST |  | 10.3 | ± | 0.9 | 0.4 | ± | 0.04 |  | 8.0 | ± | 0.7 | 0.3 | ± | 0.0 |  | 14.6 | ± | 1.4 | 0.6 | ± | 0.1 |  | 13.4 | ± | 2.2 | 0.5 | ± | 0.0 |
| PL |  | 80.8 | ± | 1.7 | 3.3 | ± | 0.2 |  | 85.2 | ± | 0.8 | 3.5 | ± | 0.3 |  | 65.3 | ± | 3.6 | 3.4 | ± | 0.4 |  | 78.5 | ± | 2.5 | 3.0 | ± | 0.4 |
| **Sum** |  |  |  |  | **4.0** | ± | **0.2** |  |  |  |  | **4.0** | ± | **0.3** |  |  |  |  | **4.5** | **±** | **0.3** |  |  |  |  | **3.7** | **±** | **0.4** |

| (b) | Zooplankton (Australia) | | | | | | | | | | | | | | | | | | Zooplankton (Mozambique) | | | | | | | | | | | |
| --- | --- | --- | --- | --- | --- | --- | --- | --- | --- | --- | --- | --- | --- | --- | --- | --- | --- | --- | --- | --- | --- | --- | --- | --- | --- | --- | --- | --- | --- | --- |
|  | Surface (n=38) | | | | | | Epipelagic (n=6) | | | | | | Demersal (n=5) | | | | | | Surface (n=8) | | | | | | Epipelagic (n=3) | | | | | |
| Lipid class | %Total lipid | | | Content (mg/g) | | | %Total lipid | | | Content (mg/g) | | | %Total lipid | | | Content (mg/g) | | | %Total lipid | | | Content (mg/g) | | | %Total lipid | | | Content (mg/g) | | |
|  |  |  |  |  |  |  |  |  |  |  |  |  |  |  |  |  |  |  |  |  |  |  |  |  |  |  |  |  |  |  |
| WE | 3.5 | ± | 0.8 | 0.3 | ± | 0.1 | 1.1 | ± | 0.4 | 0.04 | ± | 0.02 | 2.9 | ± | 1.0 | 0.1 | ± | 0.1 | 1.6 | ± | 0.5 | 0.1 | ± | 0.0 | 2.5 | ± | 0.6 | 0.2 | ± | 0.1 |
| TAG | 20.5 | ± | 1.4 | 1.7 | ± | 0.2 | 11.6 | ± | 0.8 | 0.4 | ± | 0.1 | 11.0 | ± | 2.3 | 1.0 | ± | 0.6 | 4.6 | ± | 0.5 | 0.3 | ± | 0.0 | 7.3 | ± | 0.3 | 0.5 | ± | 0.0 |
| FFA | 17.1 | ± | 0.9 | 1.3 | ± | 0.1 | 9.4 | ± | 1.0 | 0.4 | ± | 0.1 | 4.8 | ± | 0.9 | 0.4 | ± | 0.2 | 57.2 | ± | 2.1 | 3.7 | ± | 0.5 | 40.4 | ± | 1.6 | 2.6 | ± | 0.3 |
| ST | 5.5 | ± | 0.2 | 0.4 | ± | 0.02 | 6.3 | ± | 0.4 | 0.2 | ± | 0.04 | 7.1 | ± | 1.2 | 1.0 | ± | 0.5 | 6.4 | ± | 0.5 | 0.4 | ± | 0.1 | 7.1 | ± | 0.8 | 0.5 | ± | 0.1 |
| PL | 53.5 | ± | 2.1 | 4.0 | ± | 0.2 | 71.6 | ± | 2.0 | 2.7 | ± | 0.3 | 74.3 | ± | 2.7 | 9.0 | ± | 4.3 | 30.2 | ± | 1.5 | 2.0 | ± | 0.3 | 42.7 | ± | 2.7 | 2.7 | ± | 0.1 |
| **Sum** |  |  |  | **7.7** | **±** | **0.5** |  |  |  | **3.8** | **±** | **0.5** |  |  |  | **11.6** | **±** | **5.5** |  |  |  | **6.6** | **±** | **0.9** |  |  |  | **6.5** | **±** | **0.5** |

Abbreviations: WE, wax esters; TAG, triacylglycerols; FFA, free fatty acids; ST, sterols; PL, phospholipids
