# Supplementary material for: Stable Isotope and Signature Fatty Acid Analyses Suggest Reef Manta Rays Feed on Demersal Zooplankton
Source: PLoS One. 2013 Oct 22;8(10):e77152. doi: 10.1371/journal.pone.0077152 (PMC3805558; doi:10.1371/journal.pone.0077152)
Supplement: Table S2 — Results of similarity percentage analysis (SIMPER) of fatty acid data for reef manta rays and zooplankton. Fatty acids with an average contribution >8% are included. Data were not transformed prior to analysis (DOCX) [file pone.0077152.s003.docx]

Table S2. Results of similarity percentage analysis (SIMPER) of fatty acid data for reef manta rays and zooplankton. Fatty acids with an average contribution >8% are included. Data were not transformed prior to analysis

| Group | FA | Average abundance | Contribution to similarity (%) |
| --- | --- | --- | --- |
| Reef manta rays (Australia) | 18:0 | 16.1 | 18.0 |
| (86% average similarity) | 18:1ω9c | 15.4 | 16.5 |
|  | 16:0 | 13.5 | 14.6 |
|  | 22:6ω3 | 12.5 | 12.9 |
|  | 20:4ω6 | 8.7 | 8.9 |
| Reef manta rays (Australia, muscle tissue) | 18:0 | 16.0 | 17.3 |
| (90% average similarity) | 18:1ω9c | 16.0 | 17.0 |
|  | 16:0 | 13.5 | 14.1 |
|  | 22:6ω3 | 13.0 | 13.4 |
|  | 20:4ω6 | 8.7 | 8.5 |
| Reef manta rays (Australia, dermal tissue) | 18:0 | 16.7 | 20.1 |
| (81% average similarity) | 16:0 | 13.4 | 14.9 |
|  | 18:1ω9c | 12.7 | 11.6 |
|  | 20:4ω6 | 8.8 | 10.3 |
|  | 22:6ω3 | 9.8 | 9.6 |
| Reef manta rays (Mozambique) | 18:0 | 15.9 | 16.9 |
| (88% average similarity) | 18:1ω9c | 14.3 | 15.7 |
|  | 20:4ω6 | 14.2 | 14.6 |
|  | 16:0 | 12.6 | 13.5 |
|  | 22:6ω3 | 10.3 | 10.4 |
| Surface zooplankton (Australia) | 22:6ω3 | 23.4 | 23.9 |
| (85% average similarity) | 16:0 | 18.4 | 20.6 |
|  | 20:5ω3 | 13.6 | 14.7 |
|  | 16:1ω7c | 6.8 | 6.3 |
|  | 18:0 | 5.6 | 5.9 |
| Epipelagic zooplankton (Australia) | 22:6ω3 | 26.9 | 27.6 |
| (94% average similarity) | 16:0 | 19.2 | 20.2 |
|  | 20:5ω3 | 10.3 | 10.9 |
|  | 18:0 | 6.0 | 6.2 |
|  | 18:1ω9c | 4.8 | 4.8 |
| Demersal zooplankton (Australia) | 16:0 | 17.5 | 22.3 |
| (77% average similarity) | 20:5ω3 | 12.4 | 12.6 |
|  | 18:0 | 9.6 | 11.7 |
|  | 18:1ω9c | 7.0 | 8.5 |
|  | 22:6ω3 | 10.5 | 7.4 |
|  | 20:4ω6 | 5.3 | 6.3 |
| Surface zooplankton (Mozambique) | 22:6ω3 | 28.8 | 28.7 |
| (85% average similarity) | 16:0 | 19.2 | 21.2 |
|  | 20:5ω3 | 13.5 | 14.6 |
|  | 18:0 | 7.5 | 8.2 |
|  | 14:0 | 4.9 | 4.6 |
| Epipelagic zooplankton (Mozambique) | 22:6ω3 | 25.4 | 26.5 |
| (96% average similarity) | 16:0 | 22.6 | 23.7 |
|  | 20:5ω3 | 9.1 | 9.5 |
|  | 18:0 | 7.2 | 7.3 |
|  | 18:1ω9c | 7.1 | 7.3 |
| *Undinula vulgaris* | 22:6ω3 | 32.4 | 33.6 |
| (93%average similarity) | 16:0 | 16.5 | 17.2 |
|  | 20:5ω3 | 14.0 | 14.6 |
|  | 18:0 | 6.4 | 6.4 |
| Fish larvae | 22:6ω3 | 31.2 | 33.3 |
| (85% average similarity) | 16:0 | 17.6 | 17.3 |
|  | 18:0 | 11.3 | 13.0 |
|  | 18:1ω9c | 6.5 | 6.7 |
|  | 20:5ω3 | 6.7 | 5.9 |
| Shrimp-like zooplankton | 22:6ω3 | 22.6 | 24.5 |
| (85% average similarity) | 16:0 | 19.6 | 20.2 |
|  | 20:5ω3 | 16.1 | 17.4 |
|  | 18:0 | 6.6 | 6.0 |
|  | 18:1ω9c | 5.0 | 5.4 |
