# Supplementary material for: Stable Isotope and Signature Fatty Acid Analyses Suggest Reef Manta Rays Feed on Demersal Zooplankton
Source: PLoS One. 2013 Oct 22;8(10):e77152. doi: 10.1371/journal.pone.0077152 (PMC3805558; doi:10.1371/journal.pone.0077152)
Supplement: Table S3 — Fatty acid composition (% of total FA) of zooplankton taxa collected off eastern Australia. (DOCX) [file pone.0077152.s004.docx]

**Table S3**. Fatty acid composition (% of total FA) of zooplankton taxa collected off eastern Australia.

|  | Zooplankton taxa (Australia) | | | | | | | | | | | |
| --- | --- | --- | --- | --- | --- | --- | --- | --- | --- | --- | --- | --- |
|  | *Undinula vulgaris* | | | Shrimp-like zooplankton | | | Fish Larvae | | | *Subeucalanus* spp. | *Candacia ethiopica* | Eel larva |
| *n* | *n=5* | | | *n=3* | | | *n=3* | | | *n=1* | *n=1* | *n=1* |
| 14:0 | 3.8 | ± | 0.7 | 2.6 | ± | 0.3 | 1.6 | ± | 0.1 | 3.7 | 3.0 | 4.0 |
| 15:0 | 0.8 | ± | 0.1 | 0.8 | ± | 0.1 | 0.7 | ± | 0.1 | 0.3 | 0.8 | **1.2** |
| 16:0 | 16.5 | ± | 0.4 | 19.6 | ± | 2.2 | 17.6 | ± | 2.6 | 14.2 | 11.1 | 17.5 |
| 17:0 | 1.5 | ± | 0.1 | 1.4 | ± | 0.2 | 1.8 | ± | 0.2 | 0.3 | 0.8 | 1.5 |
| 18:0 | 6.3 | ± | 0.3 | 6.6 | ± | 1.5 | 11.3 | ± | 0.2 | 4.1 | 3.0 | 9.1 |
| 20:0 | 0.4 | ± | 0.0 | 0.5 | ± | 0.2 | 0.4 | ± | 0.1 | 0.7 | 0.3 | 0.4 |
| 22:0 | 0.4 | ± | 0.0 | 0.5 | ± | 0.1 | 0.4 | ± | 0.0 | 0.3 | 0.4 | 0.5 |
| 24:0 | 0.3 | ± | 0.0 | 0.3 | ± | 0.0 | 0.4 | ± | 0.0 | 0.2 | 0.3 | 0.2 |
| 26:0 | 0.1 | ± | 0.0 | 0.0 | ± | 0.0 | 0.0 | ± | 0.0 | 0.0 | 0.0 | 0.0 |
| **∑SFA** | **30.2** | **±** | **0.8** | **32.3** | **±** | **0.9** | **34.3** | **±** | **2.2** | **23.9** | **19.9** | **34.5** |
| 16:1ω7c | 5.1 | ± | 0.5 | 5.2 | ± | 1.3 | 2.5 | ± | 0.3 | 9.2 | 6.9 | 5.8 |
| 16:1ω5c | 0.2 | ± | 0.0 | 0.1 | ± | 0.0 | 0.1 | ± | 0.0 | 0.3 | 0.2 | 0.1 |
| 17:1ω8c+a17:0 | 0.2 | ± | 0.0 | 0.4 | ± | 0.1 | 0.5 | ± | 0.0 | 0.4 | 0.2 | 0.6 |
| 17:1 | 0.0 | ± | 0.0 | 0.2 | ± | 0.2 | 0.2 | ± | 0.1 | 0.0 | 0.0 | 0.2 |
| 18:1ω9c | 3.0 | ± | 0.1 | 5.0 | ± | 0.4 | 6.5 | ± | 0.8 | 2.5 | 4.2 | 4.6 |
| 18:1ω7c | 1.9 | ± | 0.1 | 2.9 | ± | 0.1 | 2.3 | ± | 0.2 | 7.1 | 2.5 | 5.0 |
| 19:1 | 0.0 | ± | 0.0 | 0.1 | ± | 0.0 | 0.0 | ± | 0.0 | 0.0 | 0.0 | 0.1 |
| 20:1ω11c | 0.1 | ± | 0.0 | 0.3 | ± | 0.3 | 0.3 | ± | 0.2 | 0.1 | 0.4 | 0.1 |
| 20:1ω9c | 0.2 | ± | 0.0 | 0.6 | ± | 0.1 | 0.4 | ± | 0.1 | 0.5 | 0.2 | 0.2 |
| 20:1ω7c | 0.1 | ± | 0.0 | 0.2 | ± | 0.0 | 0.2 | ± | 0.1 | 0.7 | 0.2 | 0.2 |
| 22:1ω11c | 0.0 | ± | 0.0 | 0.0 | ± | 0.0 | 0.0 | ± | 0.0 | 2.2 | 0.1 | 0.0 |
| 22:1ω7c | 0.2 | ± | 0.0 | 0.1 | ± | 0.0 | 0.2 | ± | 0.1 | 1.3 | 0.7 | 0.1 |
| 24:1ω11c | 0.1 | ± | 0.0 | 0.1 | ± | 0.0 | 0.0 | ± | 0.0 | 3.2 | 0.4 | 0.0 |
| 24:1ω9c | 2.4 | ± | 0.1 | 0.4 | ± | 0.1 | 0.7 | ± | 0.2 | 1.2 | 0.7 | 0.2 |
| 24:1ω7c | 0.1 | ± | 0.0 | 0.1 | ± | 0.0 | 0.0 | ± | 0.0 | 1.9 | 0.6 | 0.2 |
| 26:1ω11c | 0.3 | ± | 0.1 | 0.5 | ± | 0.2 | 1.1 | ± | 0.1 | 0.3 | 0.1 | 0.8 |
| 26:1ω7c | 0.4 | ± | 0.0 | 0.1 | ± | 0.0 | 0.1 | ± | 0.1 | 0.2 | 0.3 | 0.0 |
| **∑MUFA** | **14.6** | **±** | **0.5** | **16.8** | **±** | **0.7** | **15.5** | **±** | **1.2** | **33.9** | **18.3** | **18.7** |
| C16PUFAs | 0.2 | ± | 0.0 | 0.4 | ± | 0.1 | 0.2 | ± | 0.0 | 2.8 | 5.0 | 0.4 |
| 18:3ω6 | 0.4 | ± | 0.0 | 0.2 | ± | 0.0 | 0.1 | ± | 0.0 | 0.5 | 0.3 | 0.2 |
| 18:4ω3 | 0.8 | ± | 0.1 | 0.7 | ± | 0.1 | 0.7 | ± | 0.1 | 1.1 | 2.9 | 1.8 |
| 18:2ω6 | 1.7 | ± | 0.1 | 1.8 | ± | 0.4 | 1.7 | ± | 0.1 | 0.7 | 1.3 | 3.1 |
| 18:3ω3 | 0.7 | ± | 0.1 | 0.7 | ± | 0.1 | 0.7 | ± | 0.3 | 0.1 | 1.3 | 1.3 |
| 20:4ω6 | 1.4 | ± | 0.1 | 3.0 | ± | 0.5 | 2.7 | ± | 0.6 | 1.5 | 0.8 | 2.9 |
| 20:5ω3 | 14.0 | ± | 0.4 | 16.1 | ± | 1.4 | 6.7 | ± | 1.5 | 15.5 | 16.2 | 8.3 |
| 20:3ω6 | 0.2 | ± | 0.0 | 0.2 | ± | 0.0 | 0.2 | ± | 0.1 | 0.3 | 0.2 | 0.4 |
| 20:4ω3 | 0.4 | ± | 0.0 | 0.4 | ± | 0.0 | 0.3 | ± | 0.0 | 0.4 | 0.4 | 1.0 |
| 20:2ω6 | 0.2 | ± | 0.0 | 0.8 | ± | 0.5 | 0.3 | ± | 0.1 | 0.1 | 0.2 | 0.2 |
| 21:5ω3 | 0.1 | ± | 0.0 | 0.3 | ± | 0.0 | 0.2 | ± | 0.0 | 1.6 | 0.3 | 0.2 |
| 22:5ω6 | 0.7 | ± | 0.1 | 0.9 | ± | 0.2 | 1.1 | ± | 0.4 | 0.3 | 0.6 | 1.9 |
| 22:6ω3 | 32.4 | ± | 1.1 | 22.6 | ± | 1.9 | 31.2 | ± | 2.6 | 6.9 | 27.5 | 21.9 |
| 22:4ω6 | 0.2 | ± | 0.0 | 0.2 | ± | 0.1 | 0.3 | ± | 0.1 | 0.2 | 0.3 | 0.3 |
| 22:5ω3 | 0.5 | ± | 0.0 | 0.9 | ± | 0.2 | 2.1 | ± | 0.8 | 3.1 | 1.0 | 1.5 |
| C22PUFAs | 0.2 | ± | 0.1 | 0.2 | ± | 0.1 | 0.5 | ± | 0.1 | 0.3 | 0.1 | 0.0 |
| 28:5 | 0.4 | ± | 0.1 | 0.6 | ± | 0.2 | 0.2 | ± | 0.1 | 2.0 | 1.9 | 0.3 |
| **∑PUFA** | **54.6** | **±** | **1.0** | **50.3** | **±** | **0.1** | **49.5** | **±** | **2.1** | **42.1** | **60.9** | **46.4** |
| ω3/ω6 | 10.5 | ± | 0.6 | 6.4 | ± | 1.5 | 7.2 | ± | 2.0 | 7.8 | 12.4 | 4.03 |
| others | 0.7 | ± | 0.1 | 0.7 | ± | 0.2 | 0.9 | ± | 0.2 | 7.2 | 1.4 | 0.9 |
| ∑*iso*-SAT+*anteiso*-SAT | 0.6 | ± | 0.1 | 0.7 | ± | 0.2 | 0.6 | ± | 0.05 | 0.2 | 0.9 | 0.5 |
| EPA/DHA | 0.4 | ± | 0.02 | 0.7 | ± | 0.1 | 0.2 | ± | 0.04 | 2.2 | 0.6 | 0.4 |

∑iso-SAT+anteiso-SAT= i15:0, a15:0, i16:0, i17:0, i18:0

others=14:1ω5c, 16:1ω9c, C18PUFA, 18:1ω7t, 18:1ω5c, C20PUFA, C22PUFA, 22:1ω9c, 23:0, 24:5ω3, C24PUFA, C26PUFA, 26:2, 26:1ω9c

Abbreviations: SFA-saturated fatty acids, MUFA- monounsaturated fatty acids, PUFA-polyunsaturated fatty acids
